# Expanding Simulation Models of Emotional Understanding: The Case for Different Modalities, Body-State Simulation Prominence, and Developmental Trajectories

**DOI:** 10.3389/fpsyg.2020.00309

**Published:** 2020-03-03

**Authors:** Paddy Ross, Anthony P. Atkinson

**Affiliations:** Department of Psychology, Durham University, Durham, United Kingdom

**Keywords:** sensorimotor simulation, body-state simulation, emotion recognition, development, interoception

## Abstract

Recent models of emotion recognition suggest that when people perceive an emotional expression, they partially activate the respective emotion in themselves, providing a basis for the recognition of that emotion. Much of the focus of these models and of their evidential basis has been on sensorimotor simulation as a basis for facial expression recognition – the idea, in short, that coming to know what another feels involves simulating in your brain the motor plans and associated sensory representations engaged by the other person’s brain in producing the facial expression that you see. In this review article, we argue that simulation accounts of emotion recognition would benefit from three key extensions. First, that fuller consideration be given to simulation of bodily and vocal expressions, given that the body and voice are also important expressive channels for providing cues to another’s emotional state. Second, that simulation of other aspects of the perceived emotional state, such as changes in the autonomic nervous system and viscera, might have a more prominent role in underpinning emotion recognition than is typically proposed. Sensorimotor simulation models tend to relegate such body-state simulation to a subsidiary role, despite the plausibility of body-state simulation being able to underpin emotion recognition in the absence of typical sensorimotor simulation. Third, that simulation models of emotion recognition be extended to address how embodied processes and emotion recognition abilities develop through the lifespan. It is not currently clear how this system of sensorimotor and body-state simulation develops and in particular how this affects the development of emotion recognition ability. We review recent findings from the emotional body recognition literature and integrate recent evidence regarding the development of mimicry and interoception to significantly expand simulation models of emotion recognition.

## Background

Emotion recognition is key to successful social interactions. To date, much of the research into perceiving emotions in others has been conducted using the face, which is arguably the most salient portrayer of social signals that we possess.

**FIGURE 1:**
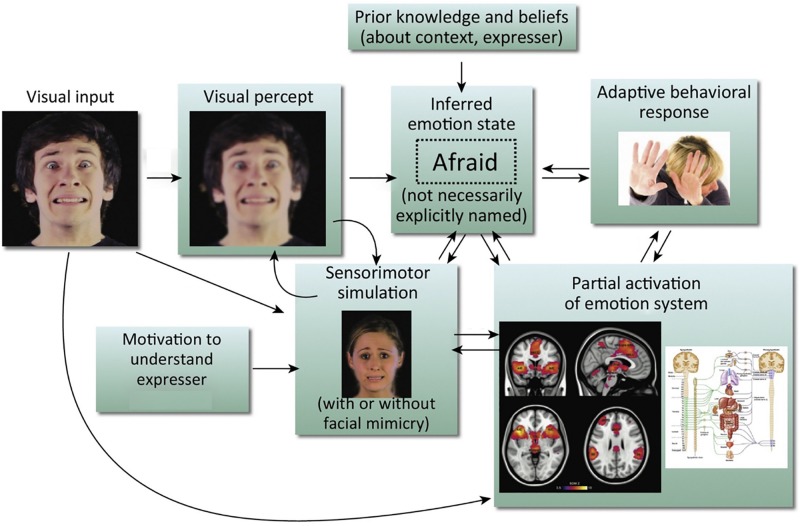
Simulation model of emotion recognition from the face. Processing of a particular facial expression triggers other components of the emotion system, leading ultimately to emotional understanding. It should be noted that arrows in this model do not imply neural modularity and specific sequential events; rather they emphasize the distributed and recursive nature of the emotion perception process. This is an amended version of a figure first published in Wood et al. (2016b). Copyright (2016) reprinted with permission from Elsevier (License No. 4626470808358).

We know that there are both visual (Ekman, 1992) and contextual cues (Kayyal et al., 2015; Xu et al., 2017) to aid accurate facial emotion recognition, but recent models also argue for the inclusion of a sensorimotor simulation system as a route to emotion recognition (Niedenthal, 2007; Wood et al., 2016a, b). The central idea of these models is that when we observe a facial expression of emotion, we can recognize the emotion by simulating the motor plans and associated sensory representations engaged by the other person’s brain in producing the expression. This can occur both with mimicry (recreating the perceived motor production of the observed facial expression), or without (see Figure 1). As we cannot directly access another’s experience, this sensorimotor simulation is part of, as Wood et al. (2016b) put it, the ‘game of prediction’ that underpins emotion recognition. These simulations are the embodied recollections of our own experiences and we are constructing the meaning of an observed emotion in part from our own prior bodily and subjective experiences of it (Niedenthal et al., 2005; Hawk et al., 2016). As such, sensorimotor simulation accounts of emotion recognition echo but reframe the James-Lange theory of emotion (Lange and Haupt, 1922; James, 1994), putting physiological states and their representation in the brain at the center not only of one’s own emotional experience but also of one’s interpretation of the other person’s emotional expression.

The result of this sensorimotor simulation is what is referred to in the literature as ‘emotional contagion,’ experiencing the feeling of another through observation (Hatfield et al., 1993; Prochazkova and Kret, 2017). However, Hatfield et al. (1993) argue that contagion is a higher-level cognitive phenomenon that can involve not only the synchronizations of facial expressions, but as mentioned above, also vocalizations, postures and the movements of another person. We already know that the body (Atkinson et al., 2004; de Gelder et al., 2010; de Gelder and Van den Stock, 2011) and the voice (Belin et al., 2008; Grosbras et al., 2018) are important expressive channels for providing cues to another’s emotional state, but to date these cues have been largely omitted in sensorimotor simulation models.

This motor matching, however, may not be the cause of the sensorimotor simulation, but rather the effect of it. Indeed, individuals can show similar expressions to another person not because they mimic the expression directly, but because the internal simulation of these emotions causes the mimicked expression (Hawk et al., 2012; Stel et al., 2016). This has even been demonstrated across expressive channels: exposure to vocal (Hawk et al., 2012) and body expressions (Magnée et al., 2007) of emotion elicits corresponding facial expressions in the observer, despite no facial expression being actually shown. The mimicry shown by subjects in these cases is therefore arguably due to the somatosensory simulation of congruent stimuli, rather than direct visual mimicry. Of course, an alternative explanation is that the act of making the expression itself generates in the observer the corresponding emotional state, but we will argue that the majority of evidence indicates that primarily motor matching is the *effect* of simulation rather than the *cause* of simulation. This line of argument is not, however, necessarily inconsistent with the reframing of the James-Lange theory that we noted above; indeed, it could be seen to be more consistent with it, giving (representations of) internal bodily states (as opposed to representations of expressive motor movements and their sensory consequences) a more central role. Of course, it is also possible that simulations of others’ internal bodily states *and* expressive changes are both causally involved in understanding their emotional expressions (which would also be consistent with the reframed James-Lange theory), a possibility to which we remain open.

This concept of emotional contagion does not even necessarily require an observer to *feel* the portrayed emotion to be able to recognize it. Recent models also include simulation that does not involve conscious awareness (Wood et al., 2016b). It is plausible that representation of changes in the autonomic nervous system and viscera (which here we will refer to as body-state simulation) is enough to underpin emotion recognition in the absence of sensorimotor simulation. We know that not only does emotional contagion occur when the stimulus is not consciously perceived, but also that unseen expressions actually evoke faster facial reactions (Tamietto et al., 2009). This unconscious body-state simulation account of emotion perception has, to date, been relegated to a somewhat subsidiary role in simulation models.

Furthermore, we know that the ability to recognize emotions from the face, body and voice follow different developmental trajectories throughout childhood and adolescence (De Sonneville et al., 2002; Herba et al., 2006; Wade et al., 2006; Tonks et al., 2007; Ross et al., 2012; Chronaki et al., 2015; Grosbras et al., 2018). This doesn’t seem to be the case in infancy, however, with evidence indicating that emotional understanding is a unified ability that develops in concert across various modalities at the same time (Missana and Grossmann, 2015). We also know from infant studies that mimicry behavior appears to precede emotional understanding and empathy development (Field et al., 1982; Meltzoff and Moore, 1983; Anisfeld, 1996; Jones, 2006). What we currently do not know, however, is how the current simulation models are affected by the developmental trajectory of embodied processes. Understanding this development could give key insights into the overall development of emotion recognition ability and may shed some light on the developmental differences across modalities.

In this review, we therefore propose three key extensions to the current sensorimotor simulation models for facial expression recognition:

(1) Fuller consideration be given to simulation of bodily and vocal expressions.
(2) The neural representation of changes in the autonomic nervous system and viscera (body-state simulation) might have a more prominent role in simulation models than currently thought.
(3) Expanding the models to address how the development of embodied processes (interoception and proprioception) affects the development of emotion recognition.

## Sensorimotor And Body-State Simulation Of Bodily And Vocal Expressions Of Emotion

Research investigating how emotional information is conveyed by whole-body cues can be traced back to the postural descriptions of Darwin (Darwin and Prodger, 1872/1998) and the body posture photographs of James (James, 1932). James noted that the subjects viewing his emotional body stimuli would imitate the posture they were looking at, and in some cases, noting emotional contagion by way of the observers expressing ‘the feeling or emotion which was attributed to the postural expression’ and that this ‘resulted from the tendency of the observer to put himself into the same situation and posture as that of the figure’ (p. 419).

This can arguably be seen as the basis for modern embodiment theory, in which individuals process the emotion information of others by activating neural states associated with their own perceptual and affective experiences (Hawk et al., 2012). In terms of the most recent work exploring sensorimotor simulation and the body, there are two main lines of enquiry. Firstly, there is work investigating simulating emotional states using facial stimuli that leads to sensorimotor simulation of the body and mimicry of some kind. Secondly, there are studies looking at various forms of mimicry due to sensorimotor simulation, but solely based on observing emotional body stimuli.

### Mimicry Response in the Body

Taking the first of these lines of enquiry, it should be noted that studies investigating body mimicry rarely do so in isolation. Instead, recent work (Wood et al., 2016b; Moody et al., 2018) describes body mimicry as a ‘spill over’ of muscle activity from the process of simulating emotions. So instead of there being a direct neural connection between visual perception and motor processes, if the emotional cue (the body for example) contains postural changes, then one would expect this ‘muscular spill over’ to extend to areas beyond the face (such as forearms/hands, etc.). Therefore, several of the studies which report body mimicry also report (or set out to investigate) facial mimicry.

Early work demonstrated mimicry in the body for non-emotional stimuli. Chartrand and Bargh (1999) showed evidence of behavioral mimicry when observing a stranger (foot shaking, face rubbing, etc.) while Berger and Hadley (1975) showed participants had larger electromyogram (EMG) activity in their arms while watching arm wrestling and in their lips when watching an actor stutter. This unconscious body mimicry was coined the ‘chameleon effect’ by Chartrand and Bargh (1999) and refers to matching one’s behavior to that of others in the environment. This may have various prosocial in-group benefits, or as Ashton-James and Levordashka (2013) argue, can be a means by which one becomes ‘liked’ by others. Either way, body mimicry appears to occur for both body and non-body stimuli alike. However, is it a mechanism by which one can obtain emotional information?

Moody and McIntosh (2011) explored this question of whether body mimicry is a general motor-matching mechanism, or if it is solely related to emotional processes. They expanded upon Berger and Hadley’s aforementioned EMG work by trying to replicate the results but in response to both emotional and non-emotional stimuli. Interestingly they found that mimicry in participants’ face actions occurs regardless of emotional content, but they did not demonstrate body mimicry to any action. They did find, however, that emotional stimuli elicited more mimicry than non-emotional stimuli.

This result rather muddies the water regarding the role of the body in mimicry, but it should be noted that this study did not include emotional body stimuli, but rather the same arm wrestling videos used by Berger and Hadley (1975).

Moody et al. (2018) followed up their 2011 partial replication study by trying to shed some light on the role of the body in the simulation and mimicry of emotional facial expressions. Here as well they used face stimuli (angry and fearful expressions), but measured EMG data from both observers’ faces and, crucially, the forearm flexor and extensor muscles of the arms. The hypothesis was that for angry stimuli, a mimicry action would involve a fist clench requiring both the flexor and extensor, whereas a raised palm in fear would only require the extensor muscle group. They found evidence that while showing the typical facial mimicry to emotional faces, subjects also demonstrated corresponding reactions over arm muscles. Importantly, this occurred despite no other emotional information being present.

These results provide convincing evidence for a sensorimotor simulation model of emotion recognition, and crucially, one in which the whole body is involved. It is still not clear, however, whether simulating the emotion at a neural level led to the physical muscular response, or whether the response comes after a more conceptual understanding of the emotion, leading to the simulation posteriori. Indeed, future research should be directed at determining the neural systems activated by these stimuli to fully understand how these whole-body simulation and mimicry processes interact with emotion recognition.

Furthermore, exploring body mimicry using dynamic emotional body stimuli would be of great interest. A replication of Moody et al. (2018) study measuring EMG activity in the face and arms, but instead using fearful and angry *body* stimuli could provide robust evidence for a sensorimotor simulation model of emotion recognition in which the whole-body response is simulated and not just the face. Rather than body mimicry being a ‘spill over effect’ of facial mimicry, it may be that using body stimuli, one finds the reverse effect.

As we have seen, motor matching in the body does occur when presented with emotional stimuli and sensorimotor simulation can be seen as a ‘whole-body’ simulation effect. Furthermore, we will later argue that the body-state simulation role in these models is not modality specific to the face. For now, however, we will review the evidence for sensorimotor simulation from body and voice stimuli.

### Studies Using Body Stimuli

It is the case that some simulation and mimicry studies have already used body stimuli, but only as a means to study facial mimicry. We know that emotions are conveyed effectively from the body (Atkinson et al., 2004; de Gelder, 2006; Ross et al., 2012) and that facial mimicry occurs when viewing body stimuli, arguably as a result of whole-body simulation, yet recent models of simulation (Wood et al., 2016b) begin with a visual input from a face stimulus. Arguably, there is no real reason that simulation models should narrow themselves in this way, and the models would perhaps benefit from widening the visual input to include body stimuli (and vocal stimuli).

Using body stimuli to study sensorimotor simulation has only entered the research agenda in the last decade, with Magnée et al. (2007) conducting the first study to directly compare EMG responses to both emotional faces and body gestures. In this initial study, Magnée and colleagues found no difference in EMG activity in the zygomaticus major (used to smile) and the corrugator supercilii (used to frown) between the two stimuli modalities. They conclude from these results that there is no evidence for the theory that the observed facial reaction is based purely on mimicry due to motor simulation. Kret et al. (2013b) support this finding showing that EMG activity from the corrugator muscle is similar across facial and body expressions of emotion. In a follow-up study, Kret et al. (2013a) also show that pupillometry response did not differ across angry/threatening bodies, scene or faces. Rather, the results are compatible with a model more akin to the recent sensorimotor simulation accounts of emotion recognition (Niedenthal, 2007; Wood et al., 2016b) where the perception of emotion (whether through the face or body) activates internal emotional states, which in turn leads to recognition and some motor response (in this case manifesting in the face).

Interestingly, the recent models of sensorimotor simulation tend to culminate in the goal of ‘recognition’ (Niedenthal, 2007; Wood et al., 2016b). However, these simulation and mimicry findings also hold when there is no conscious perception of the body stimulus (or face stimulus for that matter) at all, and thus lead to no recognition. Using a backward masking technique, Dimberg et al. (2000) showed that despite not being consciously aware of happy and angry facial expressions, participants reacted with distinct corresponding facial muscle responses to the stimuli. Tamietto and de Gelder (2008) replicated this result using backwardly masked body stimuli. They found remarkably similar results to previously reported accounts of greater zygomaticus activity in response to happy compared to fearful bodies, and greater corrugator activity for fearful compared to happy expressions.

This effect is also present in patients with blindsight. Using EMG on two patients with homonymous hemianopia Tamietto et al. (2009) found that the response of their zygomaticus and corrugator muscles was highly similar regardless of whether faces or bodies were presented to the blind visual field. Larger pupil sizes, indicative of increased emotional arousal, were also observed for fearful faces and fearful bodies relative to happy faces and bodies in both patients in both the seen and unseen conditions. Tamietto and colleagues argue that these results are more consistent with body-state simulation than with sensorimotor simulation accounts of emotion recognition (though they don’t use those exact terms), for three reasons. First, as bodily and facial expressions evoked similar facial reactions in participants, this demonstrates that patients were not simply doing ‘motor-matching.’ If this were the case, then no facial reactions should have been detected in response to bodily expressions. Secondly, as unseen face and body stimuli both triggered emotion-specific expressions in participants, motor resonance appears less automatic than emotional processing. Underlying emotion processing systems of old evolutionary origin initiate appropriate responses despite the absence of stimulus awareness. Thirdly, facial and pupillary reactions both peaked at approximately 1,100 ms from stimulus onset. Motor-matching theory would predict initial non-emotional mimicry before the emotional significance of the action is ascribed. However, Tamietto and colleagues found no indication of such sequencing between facial and pupillary responses. Rather, these reactions happened in parallel, making the motor-matching theory an unlikely explanation.

This gives further compelling evidence that facial mimicry is not simply an imitated motor response, but rather is related to some form of body-state simulation and automatic response of the emotion system. Furthermore, by showing that the response is modality independent, it provides more support for the inclusion of body stimuli into the current models of emotion recognition by sensorimotor simulation.

As above, it would also be very interesting to see if similar EMG responses as found in the face of these participants were present in the body during such studies. This would yield more evidence for a whole-body simulation model that is activated independently of conscious awareness, emotion and stimuli modality, and that gives rise to whole-body mimicry.

### Studies Using Vocal Stimuli

Facial and bodily expressions are obviously both visual stimuli. Thus, low-level processing of these stimuli occurs in similar early visual areas of the brain. Processing of auditory stimuli on the other hand, despite them containing congruent emotional information, obviously occurs very differently. The question then is, despite the differing sensory modalities, is there still evidence for the sensorimotor simulation model of emotion recognition when subjects are presented with emotional vocalizations?

In *expressing* emotional vocalizations, one’s facial activity will change to accompany the expression (Provine, 1992; Krumhuber and Scherer, 2011). Indeed, certain facial movements may be necessary for shaping the distinguishing acoustics of particular emotional utterances (Scherer, 2014). *Perceiving* positive or negative emotional vocalizations on the other hand has also been shown to elicit congruent facial mimicry (Verona et al., 2004; Magnée et al., 2007).

In terms of examining whether discrete emotional vocalizations evoke differential facial mimicry (and by extension involve sensorimotor simulation), Hawk et al. (2012) showed that facial responses were congruent when participants both produced and listened to emotional vocalizations. This simulation and consequent facial mimicry in response to vocalizations is entirely consistent with the sensorimotor simulation accounts of emotion recognition (Niedenthal, 2007; Wood et al., 2016b). Furthermore, Hawk and colleagues experiment had a condition in which participants were to reproduce the vocalization they heard before responding and a condition where they did not. When reproducing the vocalization themselves, participants were more accurate in their responses. One could interpret this finding as evidence of a more conscious and ‘active’ simulation process being utilized to aid recognition.

Furthermore, Hawk et al. (2012) also presented evidence of cross-channel simulation, whereby inhibiting participants’ facial responses modulated not only their processing of emotional vocalizations, but also their own subjective feelings of the emotion. This suggests that facial mimicry is not simply a result of motor activation or a by-product of sensorimotor simulation but is instead actively involved in the simulation of emotions from different modalities.

An interesting addition to the aural emotion recognition literature is that of emotional music. As Paquette et al. (2013) describe, recognition of the ‘basic emotions’ (happiness, sadness, anger, fear, and disgust) appears to be consistent across listeners. It is also known that emotional music can modulate brain activity in areas known to be involved in emotional processing; namely the amygdala, hypothalamus, and insula (Koelsch, 2014). Recently, Paquette et al. (2018) showed evidence for a ‘shared neural code’ for aural emotional processing across different timbres (voice, clarinet, and violin). In other words, their results support the notion of a universal acoustic code for auditory emotions that crosses aural modalities (Sachs et al., 2018). If this is the case then one potentially interesting line of enquiry could be replicating the Hawk et al. (2012) studies using emotional music rather than vocalizations. Given that musical expertise enhances the recognition of emotions from vocalizations (Lima and Castro, 2011), and the evidence of a shared neural code for aural stimuli, it is plausible that more experienced musicians may show greater simulation and consequent facial mimicry in response to emotional music. To our knowledge, there are currently no studies using music or other non-biological stimuli to explore sensorimotor simulation.

So far, we have only explored some of the behavioral evidence of this account of cross-channel simulation. However, if sensorimotor simulation of emotions from different modalities leads to a congruent motor response in the face, is there neuroimaging evidence to support this account?

### Support From Neuroimaging for the Inclusion of Body and Vocal Stimuli in Simulation Models of Emotion Recognition

There is indeed some compelling neuroimaging evidence to support these models. Reviewing the brain regions involved in the simulation of emotional states from face and body stimuli, Heberlein and Atkinson (2009) suggest that the right somatosensory cortices, amygdale, and premotor areas are involved across both modalities. The evidence suggests that the brain is modeling not only what a specific body part feels like when producing an emotional expression, but also simulating the whole body state associated with the emotion.

We know that when perceiving others’ facial expressions, activity in the somatosensory cortices correlates with activation when the perceiver generates the same expression (Van der Gaag et al., 2007). In the last decade, the role of the sensorimotor cortices in emotion recognition has widened to include other channels of expression. Using continuous theta-burst transcranial magnetic stimulation (cTMS), Banissy et al. (2010) expanded this account to include emotional voice stimuli. They found that cTMS targeting the right somatosensory cortex caused deficits in auditory emotional discrimination but not identity discrimination. Using dynamic point-light display (PLD) body stimuli and fMRI, Heberlein and Saxe (2005) showed more right somatosensory region activity when discriminating emotions compared with personality traits. This evidence suggests that the right somatosensory cortices play a role in emotion *discrimination* across modalities and not just for faces. However, how does this translate to emotion *recognition*?

There is also evidence of the somatosensory cortices showing abstract representations of different emotions. Cao et al. (2018) showed evidence of emotion-specific representation in the left post-central gyrus (lPCG) that was independent from the type of stimuli presented. Using multivariate pattern analysis (MVPA) they found that happiness could be differentiated from fear or anger in the lPCG for face, body or whole-person stimuli. This point is interesting as it was not the content of the stimuli itself that was causing these patterns of activation, but rather some secondary abstract representation of the emotion. Indeed, Peelen et al. (2010) found emotion category-specific activity patterns in the medial prefrontal cortex (MPFC) and left superior temporal sulcus (STS). This activity was modality-independent (face/body/voice) and independent of perceived emotional intensity, suggesting the representation of emotions at a more abstract level. We know that MPFC and STS are implicated in mental-state attribution and theory of mind, and the lPCG contains the somatosensory cortex and is involved in proprioception (Kato and Izumiyama, 2015), so might it be feasible that this emotion-specific encoding is a result of body-state simulation?

This account finds support from Kragel and LaBar (2016) who as well as presenting emotional facial and vocal stimuli, also took self-report ratings of participants’ own subjective emotional experience while perceiving the stimuli. They found distinct patterns of activation in the somatosensory cortex containing information sufficient to decode the perceived emotion. They also found that these patterns of activation correlated with the extent of experiential emotional mirroring across participants. These results support the account that inferring others’ emotions involves a functional role for the somatosensory cortex in representing the changes in body state associated with the perceived emotion (Damasio, 1996; Adolphs et al., 2000, 2002; Niedenthal, 2007). It also supports Hawk et al. (2012) findings of subjects’ facial responses modulating emotion recognition and one’s own emotional feeling. In other words, information related to one’s own body-state (in this case forcing a smile) contributes to the decoding of others’ emotional expressions. Given we know that emotions are associated with topographically distinct bodily sensations (Nummenmaa et al., 2014), this suggests that an emotional stimulus of any modality (i.e., face, body, voice) can lead to simulation and the resultant activation in the post-central gyrus. This can then lead to facial (or body) mimicry, adaptive behavioral responses and recognition. Therefore, sensorimotor simulation models of emotion recognition are not limited to the recognition of facial stimuli, and future models should be expanded to include these other modalities.

The majority of these results, however, show evidence for sensorimotor simulation by means of facial mimicry. We argue that there is also an important role for body-state simulation in emotion understanding. Yet sensorimotor simulation models tend to relegate body-state simulation to a subsidiary role, despite the plausibility of body-state simulation being able to underpin emotion recognition in the absence of typical sensorimotor simulation.

Of late, body-state simulation has received much less coverage than sensorimotor simulation, even though body-state simulation was prominent in the early days of simulation accounts of emotion understanding, even if not always under that name (e.g., Adolphs et al., 2000; Adolphs, 2002; Goldman and Sripada, 2005). Body-state simulation, as the name suggests, is the idea that evaluating another’s emotion involves the generation of a somatosensory representation of the body state associated with the perceived emotion. What is the evidence for the body-state simulation account of emotion recognition? We here reiterate the evidence summarized in earlier reviews (e.g., Adolphs, 2002; Gallese, 2005; Goldman and Sripada, 2005; Atkinson, 2007; Heberlein and Adolphs, 2007; Heberlein and Atkinson, 2009), and supplement it with discussion of additional, more recent evidence to argue for a more prominent role of body-state simulation in current simulation models of emotion recognition.

## The Case For A More Prominent Role For Body-State Simulation In Models Of Emotion Recognition

As we have seen above, arguably the most important evidence for the body-state simulation account of emotion recognition comes from studies demonstrating a critical role for somatosensory and insula cortices in the perception of emotions from faces, voices, and bodies. For these are regions whose primary functions are to represent changes in one’s own bodily states (i.e., interoception and proprioception). This evidence comes from studies of people with lesions to these brain areas and from studies in which transcranial magnetic stimulation (TMS) has been used to temporarily disrupt the functioning of these brain regions (however, it should be noted that insula function is not amenable to being investigated using TMS due to its location deep within the lateral sulcus, away from the scalp).

### Lesion Evidence for the Critical Involvement of Somatosensory Cortices and Insula in Emotion Recognition

**FIGURE 2:**
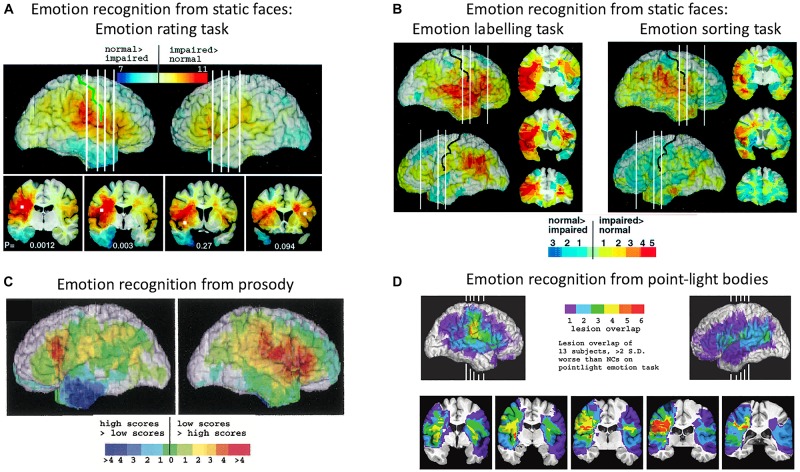
Distribution of lesion overlaps for emotion recognition tasks in studies by Adolphs and colleagues. **(A)** Distribution of lesion overlaps from all 108 subjects in Adolphs et al. (2000) study as a function of mean recognition performance on an emotion-rating task for static facial expressions. Red regions correspond to locations at which lesions resulted in impairment on the task more often than not, and blue regions correspond to locations at which lesions resulted in normal performance more often than not. **(B)** Distribution of lesion overlaps from all 108 subjects in Adolphs et al. (2000) study as a function of mean performance on tasks requiring either choosing the name of the facially expressed emotion or sorting the expressions into categories without requiring naming. Color coding as for **(A)**. **(C)** Distribution of lesion overlaps from Adolphs et al. (2002) study as a function of mean performance on an emotion-rating task for prosodic expressions, for the 33 individuals with the most abnormal ratings (red) compared with the 33 with the most normal ratings (blue). **(D)** Distribution of lesion overlaps from Heberlein et al. (2004) study for the subjects who were impaired at emotion recognition from the point-light walker stimuli (>2 SD below normal control mean). Figures **(A,B)**: Copyright 2000 Society for Neuroscience. Figure **(C)**: Copyright 2002 American Psychological Association. Figure **(D)**: Copyright 2004 MIT Press.

Adolphs et al. (2000) tested 108 patients with focal brain damage on a face emotion-rating task, then compared the overlap of brain lesion locations of the patients who were impaired on the task with the overlap of brain lesion locations of unimpaired patients. The region of maximal lesion overlap among impaired patients was in right posterior post-central gyrus, bordering on anterior supramarginal gyrus (SMG), that is, encompassing the lower sector of primary somatosensory cortex (S1) and secondary somatosensory cortex (S2). Impaired performance on the face emotion-rating task was also associated with lesions in the right insula cortex and left inferior frontal gyrus (IFG), though to a lesser extent (see Figure 2A). In two additional experiments, Adolphs and colleagues tested the same patients on a task requiring them to choose from a list of emotion words the label that best matched each face and a task requiring them to sort faces into piles according to the similarity of the emotion displayed. Impaired performance on both the emotion-naming and emotion-sorting tasks was associated with lesions in right somatosensory cortices (S1, S2), SMG and insula, as was found with the emotion-rating task. Impaired performance on the emotion-naming task was additionally associated with lesions in bilateral IFG, especially in the right hemisphere, right superior temporal gyrus, and left SMG (see Figure 2B).

A similar lesion-overlap study with a large group of patients showed that impairments in emotional prosody perception were associated with lesions to the right somatosensory cortices (S1 and S2) and insula, as well as with lesions to right IFG, right motor and premotor cortices, the left frontal operculum, principally in IFG, and bilateral frontal pole (Adolphs et al., 2002) (see Figure 2C). In a third lesion-overlap study, impaired recognition of emotions in body movements represented in point-light stimuli was associated with lesions in right somatosensory cortex, encompassing S2 and the lower sector of S1, and insula, with the region of highest overlap in right S2 (Heberlein et al., 2004) (see Figure 2D). Given that the lesion method can reveal critical roles for structures only when lesions are confined to those structures, it is significant that in both these studies, a small number of single patients with lesions restricted to right somatosensory cortex were impaired at recognizing emotions, whereas single patients with lesions that spared right somatosensory cortex were very unlikely to have impaired emotion recognition.

### TMS Evidence for the Critical Involvement of Somatosensory Cortices in Emotion Recognition

**FIGURE 3:**
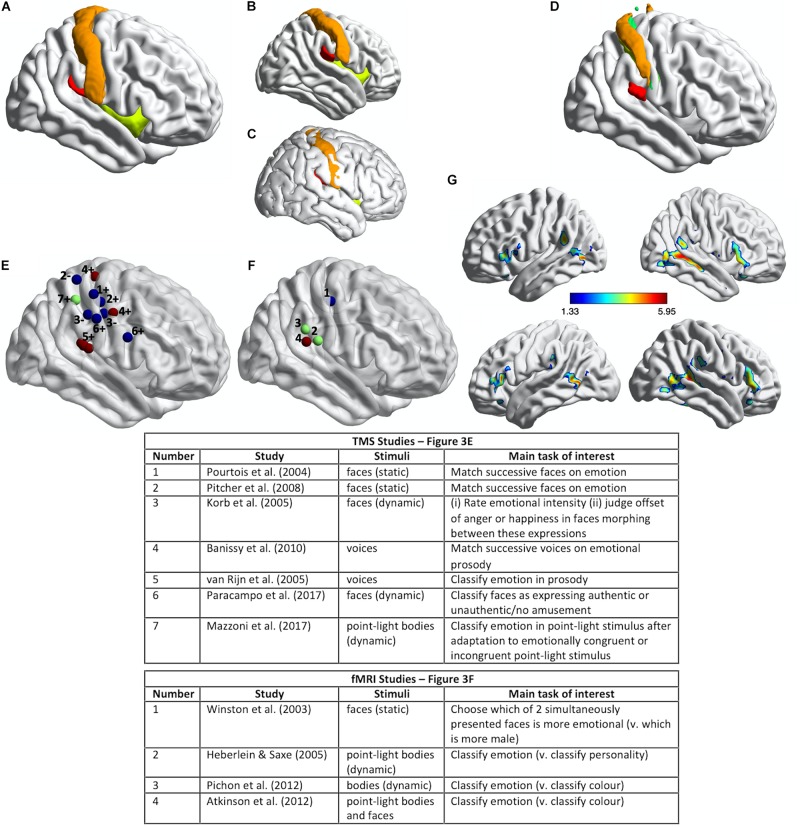
Somatosensory brain regions implicated in emotion judgments. **(A)** Renderings on a slightly inflated standard brain of post-central gyrus (orange), parietal operculum (red, corresponding approximately to S2/OP1), and insula (chartreuse yellow) in the right hemisphere, as delineated using the Harvard-Oxford Atlas (Desikan et al., 2006). **(B)** The same image as in **(A)** but rotated slightly to reveal more of S2/OP1 and insula. **(C)** The same anatomical regions rendered on a non-inflated standard brain image, to show how particularly insula and S2 are largely hidden, located away from the outermost surface of the brain (and thus skull). **(D)** Primary somatosensory cortex (S1: orange, green, yellow) and secondary somatosensory cortex (S2/OP1: red) as delineated using the probabilistic atlas from the Jülich SPM Anatomy Toolbox (Eickhoff et al., 2005). **(E)** Plotted mean coordinates, from the studies discussed in the text, of target locations for transcranial magnetic stimulation (TMS) to disrupt emotion recognition. Blue dots indicate studies that used facial expressions, red dots indicate studies that used vocal expressions, and the green dot indicates a study that used (point-light) bodily expressions. The numbers next to the dots refer to the relevant studies, as noted in the table below; ‘+’ indicates effect of TMS on emotion perception task performance; ‘−’ indicates no effect. **(F)** Plotted mean coordinates, from the studies discussed in the text, of the fMRI activation peaks in somatosensory cortices for explicit emotion judgments compared to incidental emotion processing. The blue dot indicates a study that used facial expressions, green dots indicate studies that used bodily expressions, and the pink dot indicates a study that used both bodily and facial expressions [point-light displays (PLDs)]. The numbers above the dots refer to the relevant studies, as noted in the table below. **(G)** A group statistical non-parametric map (SnPM) for emotion judgments > color judgments on point-light body and face stimuli; unpublished data from Atkinson et al. (2012). The SnPM is thresholded at *q* < 0.05, FDR-corrected (≥10 contiguous voxels). The bottom row in **(F)** is the same as the top row except for a slight rotation to reveal more of the activations in bilateral SMG/parietal operculum (including S2). For all the images in this figure, the anatomical regions, coordinate markers and fMRI activations were mapped on to a partially inflated ICBM152 standard brain in MNI space using the BrainNet Viewer software (Xia et al., 2013).

Further evidence for a critical role for right primary and secondary somatosensory cortices in the recognition of emotional expressions comes from studies using TMS. Our coverage of this evidence will highlight the heterogeneity across studies of (a) the emotion-recognition tasks used, (b) the locations over somatosensory cortices that have been targeted with TMS (indicated in Figure 3), and (c) the methods for localizing them. Given these heterogeneities, a key avenue for future research, which we discuss below, will be a more coherent and systematic attempt to tease apart somatosensory cortex contributions to emotion recognition via body-state and sensorimotor simulation.

Pourtois et al. (2004) used single-pulse TMS to interfere with the cortical processing of faces during two different perceptual discrimination decisions and found evidence for a double dissociation between emotion and eye-gaze processing. TMS applied over right S1 (Figure 3E) lengthened reaction times to match successively presented faces with respect to emotional expression, compared to TMS applied over a region of right posterior superior temporal cortex (an effect they found with fearful but not happy faces). Conversely, TMS applied over right posterior superior temporal cortex slowed matching of the same faces with respect to the direction of eye gaze, compared to TMS applied over right S1. The TMS stimulation sites were selected on the basis of the 10–20 International system of electrode placement for EEG, with the right S1 site selected as the C4 scalp location.

van Rijn et al. (2005) delivered offline 1 Hz repetitive-pulse TMS (rTMS) for 12 min to participants’ right parietal operculum/S2. This stimulation site (approximate location marked in Figure 3E) was localized on the basis of each participant’s structural MRI scan and was selected because it corresponded to the region with highest lesion-overlap associated with impaired vocal emotion recognition in the study by Adolphs et al. (2002) (Figure 2C). Immediately after TMS, the participants judged the emotion of heard sentences with respect to prosody or meaning. For the prosody task, sentences with emotionally neutral content were spoken in happy, sad, angry, and fearful tones of voice; for the emotion semantics task, sentences with happy, sad, angry, or fearful meaning were spoken in an emotionally neutral tone. Detection of emotional prosody but not of emotional meaning was significantly affected by the right somatosensory rTMS, relative to sham stimulation. Specifically, detection of ‘withdrawal emotions’ (fear plus sadness) was slowed during TMS compared to sham stimulation, whereas no effect was observed for the ‘approach emotions’ (happiness plus anger).

Pitcher et al. (2008) found that online 10 Hz rTMS applied over either right S1 (Figure 3E) or right occipital face area (OFA) disrupted the ability of observers to match successively presented faces with respect to emotional expression, as assessed with a measure of accuracy, irrespective of the particular emotion. These effects were evident relative to two control TMS conditions (stimulation over a control site and sham stimulation). The S1 stimulation site was selected to correspond to the location of peak activation for explicit vs. incidental processing of facially expressed emotions in Winston et al. (2003) study (discussed below; see Figure 3F). The same TMS over a different region of S1 (see Figure 3E), corresponding to the location of peak activation for tactile stimulation (via air puffs) of the fingers (Huang and Sereno, 2007), did not influence emotion recognition performance. Such a face-specific effect in somatosensory cortex might be considered evidence more consistent with a sensorimotor account than with a body-state simulation account of emotion discrimination, though the face-region implicated in Winston et al. (2003) and Pitcher et al. (2008) studies does not entirely fit with accurate coordinates of the face region of somatosensory cortex reported in some fMRI studies (e.g., Huang and Sereno, 2007; Eickhoff et al., 2008; Huang et al., 2012; Korb et al., 2015). Emotion discrimination accuracy was impaired relative to a matched identity discrimination task when rTMS was applied over either right OFA or right S1, while identity discrimination accuracy itself was not impaired relative to the control conditions, thus suggesting that neither region has a critical role in identity discrimination. Using double-pulse TMS delivered at different times to right OFA and right S1, this same study also demonstrated different critical periods for the involvement of these regions in emotion discrimination: right OFA’s involvement was pinpointed to a window of 60–100 ms from stimulus onset, whereas the involvement of right S1 was pinpointed to a window of 100–170 ms from stimulus onset.

Banissy et al. (2010) applied continuous theta-burst TMS (cTBS) over right S1 and, separately, over right lateral premotor cortex. cTBS is used offline (i.e., not during task performance) and has an inhibitory effect on neural and cognitive function for at least 15–20 min after its application (Wischnewski and Schutter, 2015). The right somatosensory stimulation site (Figure 3E) was localized on the basis of a previous fMRI study that contrasted touch to own face versus touch to neck (Blakemore et al., 2005). The right premotor stimulation site was localized on the basis of a previous fMRI study of non-verbal auditory emotion processing (Warren et al., 2006). Immediately prior to and again after the TMS, participants were required to judge whether pairs of successively presented, short vocalizations either expressed the same or different emotions or were voiced by the same person or different people. The stimuli comprised nonverbal vocal expressions of emotion (amusement, sadness, fear, or disgust) portrayed by four different individuals, and the same stimulus set was used in the emotion and identity discrimination tasks. Both TMS over right S1 and TMS over right lateral premotor cortex disrupted the ability to discriminate emotion but not identity from the vocal signals. This was evidenced by an increase in emotion discrimination reaction times after TMS compared to the pre-TMS baseline for the two stimulation sites of interest but not for the control stimulation site (vertex); identity discrimination reaction times, by contrast, actually decreased after TMS compared to the pre-TMS baseline for the two stimulation sites of interest but showed no difference for the control stimulation site.

Paracampo et al. (2017) used online rTMS to demonstrate critical roles for sectors of S1 and IFG in inferring authentic amusement from dynamic smiling faces. Participants in this study viewed short video clips of smiling faces, which evolved from a neutral expression to an authentic or a falsely amused smile. In one condition, the participants were asked to judge what the person truly felt (i.e., authentic amusement or no amusement) and in a control condition with the very same stimuli they were asked to judge whether a small white bar appeared above or below the person’s mouth or eyes. Paracampo and colleagues applied 12 pulses of 6 Hz rTMS, time-locked to the onset of the smile stimulus, over right ventral S1 and right IFG (see Figure 3E), as well as over right dorsomedial prefrontal cortex (dMPFC) and right temporoparietal junction (TPJ) in the neighborhood of posterior superior temporal sulcus (pSTS). The S1 site was selected as the scalp location corresponding to the region of post-central gyrus that represents tactile stimulation of the face, as indicated by several fMRI studies (Huang and Sereno, 2007; Dresel et al., 2008; Kopietz et al., 2009; Holle et al., 2013). TMS over right ventral S1 and right IFG, but not over dMPFC or TPJ/pSTS, disrupted the ability to infer amusement authenticity but did not affect performance on the control task.

The application of TMS to right somatosensory cortex does not disrupt performance on all types of emotion perception tasks, however. Korb et al. (2015) applied cTBS over regions of right S1 and primary motor cortex (M1), selected on the basis of participants’ own functional MRI localizer scans: the S1 stimulation site from tactile stimulation of the lower cheeks and the M1 stimulation site from the production of facial movement (smiling). The average coordinates of these stimulation sites are shown in Figure 3E. Korb et al. (2015) found that cTBS applied over right S1, relative to cTBS over a control site (vertex), did not affect performance either on a task requiring participants to rate the intensity of facial expressions that evolved from neutral to angry or happy, or on a task requiring participants to judge the moment at which they perceived the offset of the angry or happy expression when the dynamic faces morphed from one expression to the other. Korb et al. (2015) did, though, find that, in female but not in male participants, cTBS over either S1 or M1 reduced facial mimicry of the viewed expressions and cTBS over M1 also delayed the detection of changes from angry to happy expressions.

Although the functional roles of S1 and S2 are still not fully understood, it is clear that S2 is involved in higher-order, more integrative aspects of somatosensory processing compared to S1 (Eickhoff et al., 2005, 2010). Thus, the *a priori* case for S2’s involvement in the recognition of emotional expressions via simulation (body-state or sensorimotor) is arguably greater than that for S1. Given this and the lesion findings (discussed above) that S2 was the location of highest lesion-overlap associated with impaired emotion recognition for faces, voices and bodies, it is surprising that only one study has so far applied TMS to S2 to investigate its role in emotional expression recognition (van Rijn et al., 2005). That said, much of S2 proper (OP1 – see Figure 3) is not on the most lateral aspects of the cortex, and so application of TMS to the corresponding location on the skull could affect processing in neighboring regions of parietal and superior temporal cortex. It is thus going to be difficult to draw strong conclusions specifically about S2’s involvement from the use of TMS.

### Convergent Neuroimaging Evidence for the Involvement of Somatosensory Cortices and Insula in Emotion Recognition

An early meta-analysis of fMRI studies of emotional face processing (Fusar-Poli et al., 2009) implicated several brain regions in the explicit compared to implicit or incidental processing of those faces (i.e., when the task was to make some judgment about the emotional expression vs. a task requiring some other perceptual judgment about the emotional faces), including bilateral inferior frontal gyri, but not somatosensory or insula cortices. Nonetheless, that meta-analysis did not include Winston et al. (2003) study or subsequent studies which have shown right somatosensory cortex activation for explicit emotion judgments to faces, or studies examining the perception of emotional bodies or voices, some of which have also shown somatosensory cortex and insula involvement.

In Winston et al. (2003) fMRI study, participants attended either to the emotional content or the gender of pairs of morphed faces (morphed from a neutral face of one gender to an emotional face of the other gender). When participants attended to the emotional content (answering “Which is more emotional?”), several brain regions, including an area of right S1, was significantly more active than when participants viewed the same faces but attended to gender (“Which is more male?”). This region of S1 was the same location that Pitcher et al. (2008) subsequently targeted with TMS (see Figures 3E,F), though as noted above, this is not where several other studies have localized the somatosensory face region to be.

Heberlein and Saxe (2005) found evidence for the involvement of right S2 in emotion judgments from point-light walkers that converged with Heberlein et al. (2004) lesion overlap study. Participants in this fMRI study categorized either the emotion or the personality of the same point-light walkers. Activation for emotion judgments compared to personality judgments in right SMG corresponding to S2 (see Figure 3F) overlapped very closely the region of greatest lesion overlap that was associated with impaired emotion classification of the same point-light walkers in the 2004 study.

Other, more recent fMRI studies have reported activation in S2 and other, neighboring regions of SMG for explicit as compared to incidental processing of emotional expressions. For example, Pichon et al. (2012) had participants view short video clips of bodily movements and classify either the expressed emotion or the color of a dot that appeared briefly during the video clip. Emotion (vs. color) judgments activated regions of SMG bilaterally, including S2 (see Figure 3F), as well as several other regions, including bilateral STS, IFG, lateral occipiotemporal cortex and fusiform gyrus (see Pichon et al., 2012, Supplementary Figure 1 and Supplementary Table 1), which together comprise the so-called extended action observation network (Caspers et al., 2010; Oosterhof et al., 2013; Lingnau and Downing, 2015). Remarkably similar results were obtained by Atkinson et al. (2012), whose participants viewed short PLDs of emotional body or face movements and classified either the expressed emotion or the color of a subset of the point-lights. The activation elicited by emotion (vs. color) judgments in this latter study is shown in Figure 3G (unreported in the original publication). A similar pattern of activation was also reported by Alaerts et al. (2014), whose participants viewed successively presented pairs of PLDs of emotional body movements and indicated either whether the expression in the second display was happier, sadder, angrier or than the first or not different, or the number of dots (0, 1, 2, 3) in the second display that underwent the same color change as that of a single dot in the first display. The regions activated by emotion (vs. color) judgments included left but not right S2 (right S2 activation was evident for emotion judgments relative to a resting baseline, however).

It is notable that, with the exception of Winston et al. (2003), none of these fMRI studies reported significant activation in S1 for explicit as compared to incidental processing of the emotional expressions. (And the right S1 activation in Winston et al. (2003) study was evident only after an analysis that was focused specifically on the right primary somatosensory cortex, rather than across the whole brain). On balance, the fMRI evidence of somatosensory cortex involvement in emotion recognition (admittedly comprising only a small number of studies) points more toward S2 than S1, which reflects the findings of the lesion overlap studies of Adolphs and colleagues (Figure 2).

It is also notable that none of the fMRI studies showed activation in the insula for explicit judgments of emotion. Nonetheless, other neuroimaging studies have implicated insula cortex in emotion perception from faces, voices and bodies. For example, a recent study employing intracranial electroencephalography in patients undergoing surgery for epilepsy found a posterior-to-anterior gradient in the insula of selectivity to the emotional content of vocal stimuli, as well as an enlarged separability of the emotion types along this gradient (Zhang et al., 2019). More anterior insula, which is primarily connected to frontal and cingulate cortices and the amygdala, responded selectively to the emotional content of the prosodic stimuli, whereas posterior insula, which is primarily connected to visual, auditory, and sensorimotor cortices, responded to the acoustic properties of vocal and non-vocal stimuli regardless of emotional content. Other studies have implicated a particular and critical role for anterior insula in the perception and recognition of disgust from faces (Phillips et al., 1997, 1998; Calder et al., 2000; Adolphs et al., 2003; von dem Hagen et al., 2009; Sprengelmeyer et al., 2010) and voices (Calder et al., 2000; Sprengelmeyer et al., 2010). There is also some neuroimaging evidence for an involvement of anterior insula in the perception of emotional, especially threatening, body expressions (Pichon et al., 2009, 2012), though its involvement here does not appear to be critical for the recognition of bodily expressed disgust (Sprengelmeyer et al., 2010).

Given the findings discussed so far in this section, it is clear that there is plenty of scope for future research. One obvious avenue for such future research is in the provision of a systematic investigation of the functional roles of different sectors of somatosensory cortices and neighboring regions of parietal operculum and SMG in emotion recognition. For instance, given the heterogeneity of the locations over somatosensory cortex implicated in the TMS studies and the methods for localizing them, it is still unclear whether and how one’s own proprioceptive machinery, interoceptive machinery, or both, are critical to understanding others’ emotional states. Thus, for example, there is a need for TMS studies examining the role of somatosensory cortex in emotion understanding to localize stimulation sites on the basis of neuroimaging responses elicited by (a) first-hand emotional experience and (b) vicarious emotional experience, as well as by (c) tactile stimuli to the face or other body parts and (d) self-movements of the face or other body parts (i.e., proprioceptive responses). Moreover, ideally the sites for TMS should be individually localized on the basis of the participant’s own functional neuroimaging data and left as well as right somatosensory cortex contributions should be examined. The testing of a greater variety of emotion perception tasks within and across sensory modalities and modes of expression would also be welcome. There is also a need for studies that use connectivity methods within the context of neuroimaging and/or TMS to study how the different components of the somatosensory network interact with each other and with other networks, such as the so-called action observation and theory-of-mind networks. Finally, we note the need to examine the contribution to emotional expression understanding of other regions implicated in the lesion and/or neuroimaging work. For example, the lesion studies appear to implicate area OP4 in the right hemisphere, which is in the parietal operculum anterior to S2/OP1 and inferior to S1, especially for intensity ratings of prosodic emotions and the labeling of facial expressions, yet this region does not feature in the few fMRI studies that have contrasted emotion judgments with other judgments of the same emotional expression stimuli. OP4 is involved in sensorimotor integration, with connections to areas responsible for basic sensorimotor processing and action control (Eickhoff et al., 2005, 2010) and so would seem to be a good candidate for a role in sensorimotor simulation.

### Evidence Suggesting That Somatosensory and Insula Cortex Involvement in Emotion Recognition Goes Beyond a Proprioceptive Role

The critical involvement of right somatosensory cortex and parts of the insula in emotion recognition might be in simulating changes in the observed person’s internal bodily states, as postulated by the body-state simulation accounts, or it might be in representing the sensory consequences of simulated motor movements (specifically, the simulated production of specific facial, bodily and perhaps even vocal expressions), as postulated by sensorimotor simulation accounts. This amounts to a difference between a more body-part specific, proprioceptive role and a more global, somatovisceral role for somatosensory and insula regions in emotion recognition. In this subsection, we review evidence that somatosensory and insula cortices have a more global, somatovisceral role in emotion perception and recognition.

Certainly, there is evidence implicating S1, S2 and insula in emotional experience, which goes beyond or does not obviously involve the representation and integration of proprioceptive and tactile information. For example, using Positron emission tomography (PET), Damasio et al. (2000) found changes in activation of S2 and insula, as well as in other regions that receive signals from the body, including cingulate cortex, brainstem nuclei and hypothalamus, during the experience of sadness, happiness, anger and fear relative to emotionally neutral experience (induced via the recall of emotionally powerful or emotionally neutral personal episodes). Interestingly, activity increased (relative to neutral) in S2 when the participants reflected on happy situations, whereas it decreased when they reflected on sad situations. In an fMRI study, somatosensory cortex and ventral posterior insula activation correlated with the intensity but not the pleasantness of warm and cold thermal stimuli delivered to the hand (Rolls et al., 2008). Another fMRI study reported that the magnitude of subjectively experienced disgust correlated with anterior insula activity (Harrison et al., 2010). This latter finding converges with prior work with intracranial recordings and stimulation (Krolak-Salmon et al., 2003) and with a lesion patient (Calder et al., 2000), that anterior insula, perhaps in concert with basal ganglia structures, plays a critical role in the experience as well as – as we noted above – in the perception of disgust. In another lesion study, Johnsen et al. (2009) found that damage to right somatosensory cortex impaired the ability to experience emotions from music, as measured by self-rated feelings, but left autonomic arousal to that music, as measured by skin-conductance response, unaffected. The lesions likely included both S1 and S2, though this was not reported. The ability of the patients with right somatosensory cortex lesions to recognize the intended emotion in music was not significantly impaired, however, showing only a slight reduction compared to a non-brain damaged control group.

In an fMRI study, Straube and Miltner (2011) presented neurologically healthy participants with aversive and neutral pictures in each of four conditions intended to parametrically vary the participant’s attention to their own emotional involvement. The results showed a parametric increase of activation specifically in the right posterior insula and right S1 and S2 with increasing personal emotional involvement – from little or no engagement with the content of the pictures, through a focus on the pictures but not explicitly on their emotional content, a focus on the emotional significance of the picture but not explicitly on their own emotional response, to a focus on their own emotional experience (whether they experienced the picture as unpleasant or not unpleasant). “These findings,” Straube and Miltner claim, “are in accordance with theories suggesting a crucial role of the perception of bodily states for emotional experiences” (p. 2534).

In another fMRI study, Sel et al. (2014) found that emotional (happy or fearful) compared to neutral faces enhanced early somatosensory activity (145–185 ms after face onset), as measured with EEG, when participants were attending to the face’s emotional expression (probed on 20% of trials to judge whether the face was fearful or happy), but not when they were attending to the face’s gender (probed on 20% of trials to judge whether the face was female or male). Importantly, Sel et al. (2014) showed that this effect manifested as somatosensory-evoked activity, distinct from visual-evoked activity, during the critical period for somatosensory cortex involvement in facial emotion recognition identified by Pitcher et al. (2008). They did this by presenting tactile stimulation both to the participant’s face (left cheek) and to their (left) finger during some trials at 105 ms from face image onset, and then subtracting for each participant the relevant evoked potentials for a visual-only face condition from the evoked potentials for the same participant for each of the visual-tactile face conditions. Moreover, these effects (occurring 40–80 ms from tactile stimuli onset) were source localized within S1, S2, and associative somatosensory cortex (Brodmann area 5, which is immediately posterior to post-central gyrus in superior parietal cortex). (Note that it is more difficult to reliably and accurately source localize EEG signals from deeper structures such as the insula). These findings suggest that somatosensory cortex involvement in facial emotion perception reflects the engagement of processes that include more widespread, non-facial as well as facial somatosensory states associated with the viewed emotion, one of which includes changes in facial muscle activity (but is much less likely to include finger muscle activity). These results are thus more in-line with the body-state simulation accounts than with the sensorimotor simulation accounts of emotion recognition. Nonetheless, it is possible that processes implementing sensorimotor simulation are engaged particularly when the task is to identify or categorize the perceived expression, but that such processes were engaged little or not at all by Sel et al. (2014) paradigm because participants were required to categorize the expressed emotion on only 20% of trials.

As we briefly discussed in section “Sensorimotor and Body-State Simulation of Bodily and Vocal Expressions of Emotion,” Kragel and LaBar (2016) used fMRI with MVPA to reveal activation patterns in right post-central gyrus (S1) and bilateral insula, as well as in inferior frontal gyrus, medial orbitofrontal cortex and fusiform gyrus, that contained information sufficient to decode emotions conveyed in facial and vocal expressions (anger, fear, happiness, sadness, surprise, and emotionally neutral). Notably, Kragel and LaBar also showed that the emotion-specific patterns of fMRI activity (collapsed over stimulus modality) within right post-central gyrus, but not within any of their other regions of interest, correlated with participant self-reports of subjective experience generated by the facial and vocal expressions. Furthermore, these activation patterns (collapsed over stimulus modality) exhibited somatotopic organization, to the extent that greater activation in inferior regions of the post-central gyrus predicted perception of emotions whose facial expressions contain more distinguishing information in the lower portions of the face (happiness and surprise), whereas greater activation in superior regions of the post-central gyrus predicted perception of emotions whose facial expressions contain more distinguishing information in upper portions of the face (anger and fear). It is also notable that, although activity patterns in right *pre*central gyrus (primary motor cortex) predicted the emotional content of the stimuli, these activity patterns did not correlate with participant self-reports of subjective experience generated by those stimuli; moreover, activity patterns in left precentral gyrus neither predicted the emotional content of the stimuli nor were correlated with self-reports of subjective experience. This suggests that sensorimotor simulation is not likely responsible for primary somatosensory cortex’s role in linking perception of emotional expressions with subjective emotional experience, at least in the context of a task requiring self-reports of emotional feeling elicited by those emotional expressions. But as Kragel and LaBar acknowledge, “future work more precisely monitoring facial muscle activity will be necessary to definitively resolve this issue” (p. 10). And it leaves open the possibility that explicit identification or categorisation of the expressed emotion does (at least sometimes) engage processes of sensorimotor simulation.

So far in this subsection we have seen that somatosensory cortex, particularly S2 but perhaps also S1, and insula are engaged by a range of affective experience and to different extents depending on the intensity and/or degree of personal significance – an involvement that, particularly for S2 and insula, might include but likely goes beyond the representation and integration of proprioceptive and tactile information related to that experience. Single-case lesion evidence suggests that the insula does not directly contribute to the conscious experience of emotions, however. Damasio et al. (2013) report the case of a patient with entire destruction of the insulae bilaterally who showed intact and wide-ranging experience of emotional and bodily states, including happiness, sadness, apprehension, irritation, caring, compassion, pain, pleasure, itch, tickle, hunger, and thirst. He also behaved in ways consonant with these states when experiencing them. The authors suggest that subcortical structures are the primary neural substrate of emotional and bodily feelings, with those basic feeling states being remapped and integrated in cortical structures such as the insula. The function of the insula in emotional experience would thus be, they suggest, in underpinning higher-level processes that relate basic feeling states to cognitive processes (e.g., decision making, episodic memory, language). In another case study, Feinstein et al. (2016) studied a patient with extensive bilateral lesions to the insula, anterior cingulate and amygdala, which are key structures activated when subjects experience pain, and found he had preserved emotional awareness of pain. Feinstein et al. (2016) suggest that these regions may be more important for the regulation of pain than for directly underpinning conscious experience of pain.

The central claim of the body-state simulation proposal is that brain regions such as the somatosensory cortices and insula are also engaged by (and represent) other people’s emotional experience, and in particular that they represent the changes in body state associated with the perceived emotion. A key part of the evidence for this claim comes from studies demonstrating common neural substrates for perceiving an emotion in another and experiencing that same emotion for oneself. For example, there is evidence that parts of anterior insula and adjacent frontal operculum are activated both by the perception of someone else’s facially expressed disgust and by the feeling of disgust for oneself (Wicker et al., 2003). Such activation overlap in the insula and adjacent frontal operculum for first-hand and vicarious experience is not limited to disgust, however; it has also been demonstrated for gustatory-related pleasant experience, for example (Jabbi et al., 2007) and, along with a small number of other regions, particularly anterior cingulate cortex, pain (e.g., Singer et al., 2004; Jackson et al., 2005; Lamm et al., 2011; Krishnan et al., 2016).

Does this common activation for felt and perceived pain or disgust (or other emotions) indicate a common neural representation? More recent research provides evidence that, although there may be weak shared multivoxel pattern information across a small number of brain regions, including anterior insula and anterior cingulate (Corradi-Dell’Acqua et al., 2011, 2016), local patterns of activation in these regions do not accurately predict either vicarious or first-hand experience of pain (Krishnan et al., 2016) – a finding that has yet to be tested for disgust or other emotions. Instead, Krishnan et al. (2016) findings indicate that first-hand (somatic) pain and vicarious pain are represented across distinct brain networks, with first-hand (somatic) pain encoded largely within anterior insula, dorsal posterior insula, anterior cingulate and S2, and vicarious pain largely within portions of the dMPFC, amygdala, posterior cingulate, and TPJ.

Thus, although common activation is observed in anterior insula and anterior cingulate for first-hand and vicarious pain, this does not appear to be related to shared pain experience. Indeed, Corradi-Dell’Acqua et al. (2016) found similarities in activity patterns in left anterior insula and mid-anterior cingulate between pain, disgust, and fairness as well as between first-hand and vicarious experiences of those states (Activity patterns in right anterior insula, on the other hand, were specific to the modality and the subject of the experience).

Given the findings and interpretations discussed in this section, it is evident that there are still many outstanding questions for future research to answer. For example: what aspects of another’s expressed emotion is represented or simulated in somatosensory cortices and insula? What are the processes underpinning body-state and sensorimotor simulation? We believe that a promising theoretical approach in which to address these questions is provided by the predictive coding framework, which has recently been applied to the related questions of how we come to understand others’ actions and mental states and how we come to share their bodily sensations (Kilner et al., 2007; Zentgraf et al., 2011; Gu et al., 2013; Koster-Hale and Saxe, 2013; Barrett and Simmons, 2015; Ishida et al., 2015; Ondobaka et al., 2017).

## The Development Of Simulation Models Of Emotion Recogniton

We have so far argued for the inclusion of body and vocal stimuli in these simulation models and that body-state simulation may have a more prominent role than currently thought. In the last section of this review, we will look at sensorimotor and body-state simulation models from a developmental point of view. Specifically, given the differences in the developmental trajectories of emotion recognition across modalities, what does current work on the development of emotion recognition and embodied processes in infants and children tell us about the nature of sensorimotor and body-state simulation? As we have argued previously, research into emotion recognition from vocal and body cues has received substantially less attention than work examining faces. Here, then, we must also consider these modalities from a developmental point of view.

### The Origin of Emotional Understanding

From very early on, infants are highly attentive to social stimuli. The information that they can detect from these stimuli, however, changes with the perceptual system. For example, an infant’s visual acuity improves over the first 6 months of life from a position of only discerning blurry features in the first few months, to detecting relational information (smiling/raised eyebrows) by 6 months of age (Gwiazda et al., 1989). Indeed, by 5 months infants can form categories of happy facial expressions, with a broader range of categorical expression detection becoming apparent by 7 months of age (Grossmann, 2010). Crucially, by the end of their first year, infants also use facial expressions of others to guide their behaviors in uncertain situations, as evidenced by the classic ‘visual cliff’ paradigm (Sorce et al., 1985). This implies some more complex understanding of the emotion, or at least of the context in which one might display such an emotion. Alternatively, might this be evidence of early simulation, whereby the mother’s happy emotion elicits the same feeling in the infant, causing the feeling of safety in the context of the visual cliff task?

Emotion recognition also has a complex developmental trajectory, with studies suggesting that recognition of facial emotion is not adult-like until approximately 11 years of age (Tonks et al., 2007; Gao and Maurer, 2010; Chronaki et al., 2015), bodily emotions at approximately 8 years of age (Boone and Cunningham, 1998; Lagerlof and Djerf, 2009; Ross et al., 2012) and vocal emotion recognition ability still developing into adolescence (Chronaki et al., 2015; Grosbras et al., 2018). Might these differing trajectories be a product of differential development in simulation abilities?

Alternatively, could these differences be attributed to a lack of *articulation* ability? Pre-school (approximately 3 years old) children have been shown to accurately identify happy and angry facial expressions, with sad, disgusted and fearful faces proving harder for them to label (Kujawa et al., 2014). This of course could be a product of limited language capabilities and task difficulty, but if ‘emotion recognition’ is to be the end-point for sensorimotor simulation models, then this is problematic for the inclusion of young children who may recognize the emotion, but not have the capability to conceptualize it or to articulate an appropriate response. Furthermore, there is a distinction between emotion *discrimination* [as one might find in infant event-related potential (ERP) studies] and emotion *recognition* (arguably what intermodal matching tasks are examining). Indeed, emotion *recognition* is also arguably synonymous with emotional *labeling*, so perhaps a distinction could be made explicit in future simulation models by referring instead to emotional *understanding*. This would allow for the inclusion of infants and children into the models and would encompass discrimination, recognition and labeling.

In terms of neural processes at a young age, the focus of emotion perception (or *understanding*) research in infancy has been facial expressions (review in Leppänen and Nelson, 2009). The main findings of this work have shown that infants can discriminate between positive and negative facial emotions by 7 months of age (Nelson et al., 1979) and that the neural processes associated with the perceptual encoding of faces are modulated by emotional facial expressions (Nelson and De Haan, 1996; Leppänen et al., 2007). However, recent ERP evidence has shown that 8-month-olds discriminate between facial expressions of fear and happiness, but only when presented in the context of a congruent body (Rajhans et al., 2016). This work indicates the speed with which emotional understanding abilities come online, and also the importance of other emotional modalities in the development of emotional understanding.

The development of the behavioral responses and neural correlates of emotion recognition ability from the body is work that has gained traction only in the last 5 years. In the first study to explore the development of the ability to respond to emotional information carried in body motion, Zieber et al. (2014a) used full-light videos of emotional body expressions to show that 6.5-month-old infants showed a visual preference for happy over neutral bodies. Infants could also match emotional information from these dynamic body movements with emotional vocalizations, looking longer at congruent body-voice emotional pairings compared with incongruent information. The authors replicated these results using static bodies in a body-vocalization matching task, again finding that 6.5-month-olds could match the stimuli but the 3.5-month-olds could not (Zieber et al., 2014b).

Investigating the neural processes underpinning the development of emotion recognition from the body, Missana et al. (2014a) presented 4 and 8-month-olds with upright and inverted happy and fearful dynamic PLDs while taking ERP measures. They found that, similar to work using facial and vocal emotional expressions (Nelson and De Haan, 1996; Grossmann et al., 2005), by 8 months of age infants showed a neural discrimination between fearful and happy body movements that wasn’t apparent at 4 months. Missana et al. (2014b) further replicated this result using static body stimuli with 8-month-old infants.

The development of emotional prosody also follows a similar trajectory in the first years of life. A new-born prefers their own mother’s voice over that of a stranger’s (DeCasper and Fifer, 1980), but by 4 months can make fine discriminations among different human speech sounds and characteristics (Caron et al., 1988). In terms of emotional understanding, ERP data suggests that 7-month-old infants allocate more attention to angry rather than happy or neutral voices (Grossmann et al., 2005). An EEG study with 8-month-old infants showed that hearing another infant laugh produced enhanced positivity around 300 ms while hearing them cry produced increased negativity around 200 ms (Missana et al., 2017).

Similar to the visual cliff example in face research, by 12 months of age, children were also found to modulate their exploratory behavior based on linguistic cues (Mumme and Fernald, 2003). After observing an actor responding positively to an object, infants touched the object more than a distractor object, and avoided the object if the actor reacted negatively. Furthermore, infants showed more facial expressions of negative affect during the negative-emotion trials, suggesting emotional contagion, whereby the actor’s negative reactions had an effect on the infants’ emotional state. This then could have influenced their response to the object, by means of simulating how the actor felt toward it.

This evidence suggests that in the first year of life, emotional understanding appears to be a unified ability that develops in parallel across various modalities (face, bodies, and voice) (Missana and Grossmann, 2015). However, is there evidence of parallel development of simulation by way of motor response in line with adult studies?

### Evidence of Motor Matching in Children

Imitation to behavioral stimuli appears to develop throughout most of the second year of life (Jones, 2007). Furthermore, mimicry of behavioral cues is only one form of imitation, and as Thelen and Smith (1996) argue, its drawn out developmental trajectory suggests that it is the product of a large number of component kinds of motor, cognitive, and social knowledge that each has its own developmental course.

There is evidence of very early emotional contagion in infants that would suggest some form of affect sharing in the first year of life. Infants have been shown to be able to differentiate between their own cry and that of another whether awake or asleep (Dondi et al., 1999) and Geangu et al. (2010) showed that playing another infant’s cry caused increased vocal and facial expressions of distress in infants as young as 1-month old. These type of reactions have also been shown to be accompanied by physiological body-state changes such as changes in sucking and heart rates (Field et al., 2007). Interestingly, this mirrors the work in adults showing that facial expressions of emotion were triggered by auditory stimuli (Magnée et al., 2007). Could this not then be deemed emotion recognition? Obviously, there is no labeling to communicate the meaning of the emotion, but the physiological response hints at an emotion processing system that is creating both sensorimotor and body-state simulation, nonetheless. Therefore, one might argue that if it is not emotion *recognition per se*, it is certainly evidence of emotional *understanding*.

Further evidence of this simulation in children comes from EMG studies measuring rapid facial responses (RFRs) to emotional stimuli. Using infants, Kaiser et al. (2017) found that there was no evidence of facial EMG response to emotional faces in 4-month-olds, but for 7-month-olds, there was evidence of selective activations in the relevant regions for happy and fearful faces. Datyner et al. (2017) showed similar results in 7-month-olds viewing happy and angry facial expressions. Interestingly, Isomura and Nakano (2016) showed that infants aged 4–5 months showed increased corrugator EMG response to audio-visual crying and increased zygomaticus EMG response to audio-visual laughing, but no clear increase in response to unimodal emotional stimuli (faces or vocalizations individually). There is evidence that at this age, infants can discriminate happy, sad and angry emotions from bimodal audio-visual stimuli, but that sensitivity to unimodal auditory stimuli emerges at 5 months, and visual stimuli at 7 months (Flom and Bahrick, 2007).

There is the suggestion, however, that these RFRs are merely infants showing appropriate emotion responses to the stimuli rather than mimicking the facial expression observed. In slightly older infants (9–10 month olds), Hashiya et al. (2019) showed that infants’ facial responses increased with repeated observation of dynamic morphed faces. This suggests that infants of that age at least are not performing this task based on a purely mimicking mechanism of action matching, but rather may support a more complex affect recognition system (Hess and Fischer, 2014).

In pre-school children (3 year olds), Geangu et al. (2016) looked at EMG response to emotional bodies and faces. Similarly, they found increased activation of the zygomaticus major and decreased activation in the frontalis medialis in response to happy faces and the opposite effect for angry faces. This very much mirrors the results we have previously discussed in adults. They did not, however, replicate the cross-modality effect of Magnée et al. (2007) and Tamietto and de Gelder (2008) when presenting the emotional bodies. One explanation the authors give for this is the relative inability of children to link body expressions with the event most likely to cause such an affect. If this were the case then there would likely not be sensorimotor simulation of the body affect, as the child would have no contextual reference for such an experience.

An alternative explanation could simply be the use of static bodies. As the emotion of a body can be closely linked with the action it is performing (Atkinson et al., 2004; de Gelder, 2006, 2009), dynamic rather than static body expressions might be more likely to elicit facial EMG responses that distinguish between emotions. Indeed, one recent study provides evidence that facial EMG responses can distinguish between emotions expressed in body movements even in 11-month old infants. Addabbo et al. (2019) presented infants with video clips of adult upper bodies, from the neck to the waist, with the depicted person moving objects across a table into a box with either an angry or happy movement. They reported increased EMG responses over the zygomaticus (smiling) muscle and decreased EMG responses over the corrugator (frowning) muscle when the infants observed happy arm movements and the converse – i.e., decreased EMG responses over the zygomaticus muscle and increased EMG responses over the corrugator muscle – when the infants observed angry arm movements.

In older children (6–7 years old), Deschamps et al. (2015) showed similar facial EMG responses as previously described to emotional faces. Interestingly, they also found no significant difference in facial EMG between healthy controls and patients with ASD. Previous studies have shown evidence of similar voluntary mimicry of happy and angry faces between these groups, but also report a lack of automatic mimicry in ASD participants (McIntosh et al., 2006).

In many of these studies, children are not yet ‘adult-like’ in their emotion recognition ability: recent fMRI evidence showed that when presented with dynamic emotional bodies, the size and strength of activity in the body-selective areas of the brain were still developing through childhood and adolescence. However, the emotion modulation of these areas was already adult-like in children as young as 6 years of age (Ross et al., 2019). This would suggest that the ability to discern different movement profiles and action intentions continues to develop through experience, but mirroring the EMG work, the emotional content of these stimuli is processed no differently in children than in adults. Therefore, differences in recognition may be either due to an immature ability to accurately articulate the presented emotion, or a lack of experience, thus indicating a lack of appropriate sensorimotor and body-state simulation. One potential cause of this lack of appropriate simulation ability may be immature interoceptive and proprioceptive abilities.

### The Development of Embodied Processes

In the following section we will use ‘embodied processes’ to mean interoception and proprioception unless we are referring to one or the other explicitly. We will, however, largely focus on the development of interoception, as it is a key part of body-state simulation. The development of proprioception is of course important, especially in relation to the sensorimotor simulation proposal, but there are, to our knowledge, currently no studies that have looked at a link between the development of proprioception and emotional understanding.

A sense of embodiment (the feeling of inhabiting a body) is crucial for self-experience and self-recognition (Carruthers, 2008; Kilteni et al., 2012). Craig (2009) argues that interoception is fundamental to our subjective feeling states while proprioception is crucial for the neural control of movement and sense of self (Aman et al., 2015). However, as these abilities develop, how does this affect emotional understanding through the simulation model? Is there a causal relationship between embodied processes and emotional understanding by simulation? Or, in other words, does one need suitably developed interoception and proprioception in oneself before one can simulate the emotions of others? We leave these as open questions but review the recent literature picture to date.

Studies investigating interoception have mostly quantified interceptive sensitivity by measuring a persons’ ability to perceive their own heartbeat (Schandry, 1981; Ainley et al., 2012). Here a subject is asked to silently count their heartbeats while their actual heartbeats are recorded, allowing a sensitivity score to be generated. This is arguably a measure of conscious body-state awareness; indeed, there is an issue of how one would measure unconscious body-state awareness that will be explored later. Greater cardiac awareness (heartbeat sensitivity) has been linked with better sympathetic reactivity during mental stress and more subjective arousal during emotional picture viewing (Herbert et al., 2010). Heartbeat detection has been shown to be associated with the intensity of emotional experience (Wiens et al., 2000) as well as predicting performance on emotional memory tasks (Pollatos and Schandry, 2008).

There is also evidence of interoceptive facilitation of the judgment of emotional faces (Gray et al., 2012; Garfinkel et al., 2014) while in a study using individuals with Autism Spectrum Conditions (ASC), Mulcahy et al. (2019) linked the deficit in the recognition of affective prosody in this group with reduced interoceptive awareness.

Perhaps unsurprisingly, in heartbeat tasks using children, miscounting or cognitive overload means that error rates tend to be much higher than adult studies. In one study, for example, 12% of children were unable to detect any heartbeat at all, while only 9% were classified as having good heartbeat perception (Eley et al., 2004). A more recent large scale study (∼1300 6–12 year old children) measuring cardiac awareness and interpersonal emotional intelligence found a positive relationship between the two measures, suggesting that bodily sensitivity and interoceptive development may have a relationship with emotional understanding (Koch and Pollatos, 2014). Additionally, by shortening the intervals for heartbeat tracking, they were able to obtain similar cardiac awareness measures in children as in adults (only 5% were unable to detect any heartbeat).

A recent study by Schaan et al. (2019) examined the relationship between cardiac awareness and emotion recognition in even younger children (4–6 year olds) using the ‘Jumping Jack Paradigm’ (JJP). In this task, children were asked to do jumping jacks for 10 s and then indicate, by means of different sized circles, how fast their heart was beating prior to and following the exercise. This alleviates the issue of arithmetic ability hindering the measure of cardiac awareness. However, they found that interoceptive accuracy did not account for the variance in emotion recognition accuracy scores when labeling emotional faces. This could be due to the method of measuring cardiac awareness being too vague, or, due to the age of the children, emotion recognition labeling may just be quite poor given they included more complex and ambiguous emotions such as surprise and disgust. This research raises an interesting question for the literature; how does one accurately measure interoceptive ability in young children?

Using 5-month-old infants, Maister et al. (2017) used a paradigm in which an animated character moved either in synchrony or asynchrony to the infant’s heartbeat. They found that infants spent longer looking at the asynchronous character, indicating an awareness of their own interoceptive signals. This study also utilized a type of ERP measured by EEG, termed the ‘heartbeat-evoked potential’ (HEP). This potential is derived by averaging EEG signal that is time-locked to the subject’s heartbeat and is considered to reflect unconscious cortical processing of cardiac activity. Previous work has shown the HEP to be correlated with self-rated empathy scores in adults (Fukushima et al., 2011) as well as being modulated by the perception of sad faces (Kim et al., 2019). This evidence suggests that unconscious cardiac monitoring in the brain is involved in processing the emotional states of others. Maister et al. (2017) found that HEP response increased in those infants that showed a strong preference for the asynchronous character in the heartbeat task. Furthermore, HEP response increased when infants viewed fearful or angry face video clips, suggesting that infants’ brains are monitoring their hearts more closely when confronted with negative emotions.

This technique may be the answer to measuring interoception in young children. If it could be combined with the motor matching EMG studies already reviewed, or the emotion recognition and discrimination tasks in infancy and childhood, it could provide intriguing new evidence detailing how these systems develop. It would allow the direct comparison of the development of emotional understanding ability and simulation by means of unconscious interoceptive processes, greatly improving our understanding of the development of sensorimotor and body-state simulation as a basis for emotion recognition.

## Concluding Remarks

In conclusion, the current review argues for three key extensions to the simulation models of emotional understanding. Firstly, by including body and vocal stimuli in the model as potential visual and auditory inputs, respectively, and body mimicry as a product of simulation, the models will better reflect the multimodal world in which we live. Furthermore, it will allow for a more complex theory of modality interactivity and a fuller understanding of simulation as a means of emotional understanding. Secondly, we argue that given the converging neuroimaging, brain stimulation and lesion evidence for the role of somatosensory and insula cortices in emotion recognition, body-state simulation should be given more prominence in simulation models. Yet there is still more work to be done to elucidate exactly what and how distinct regions of somatosensory and insula cortices contribute to emotion understanding via body-state simulation, sensorimotor simulation, or both. Finally, by expanding simulation models to include the development of interoception and proprioception in parallel with the development of emotional understanding, we will be able to explore the origins of simulation, and in turn learn more about the cognitive processes underlying this mode of emotional understanding.

## Author Contributions

PR and AA both wrote and edited the manuscript.

## Conflict of Interest

The authors declare that the research was conducted in the absence of any commercial or financial relationships that could be construed as a potential conflict of interest.
